# Diagnostic Accuracy of Quantitative PCR (Xpert MTB/RIF) for Tuberculous Meningitis in a High Burden Setting: A Prospective Study

**DOI:** 10.1371/journal.pmed.1001536

**Published:** 2013-10-22

**Authors:** Vinod B. Patel, Grant Theron, Laura Lenders, Brian Matinyena, Cathy Connolly, Ravesh Singh, Yacoob Coovadia, Thumbi Ndung'u, Keertan Dheda

**Affiliations:** 1 Department of Neurology, University of KwaZulu-Natal, Durban, South Africa; 2 Lung Infection and Immunity Unit, Division of Pulmonology & UCT Lung Institute, Department of Medicine, University of Cape Town, Cape Town, South Africa; 3 Biostatistics Unit, Medical Research Council, Durban, South Africa; 4 KwaZulu-Natal Research Institute for Tuberculosis and HIV, Nelson R Mandela School of Medicine, University of KwaZulu-Natal, Durban, South Africa; 5 HIV Pathogenesis Programme, Doris Duke Medical Research Institute, Nelson R Mandela School of Medicine, University of KwaZulu-Natal, Durban, South Africa; 6 Department of Medical Microbiology, University of KwaZulu-Natal, Durban, South Africa; 7 National Health Laboratory Service, Durban, South Africa; 8 Institute of Infectious Disease and Molecular Medicine, University of Cape Town, Cape Town, South Africa; University of Minnesota, United States of America

## Abstract

**Background:**

Tuberculous meningitis (TBM) is difficult to diagnose promptly. The utility of the Xpert MTB/RIF test for the diagnosis of TBM remains unclear, and the effect of host- and sample-related factors on test performance is unknown. This study sought to evaluate the sensitivity and specificity of Xpert MTB/RIF for the diagnosis of TBM.

**Methods and Findings:**

235 South-African patients with a meningeal-like illness were categorised as having definite (culture or Amplicor PCR positive), probable (anti-TBM treatment initiated but microbiological confirmation lacking), or non-TBM. Xpert MTB/RIF accuracy was evaluated using 1 ml of uncentrifuged and, when available, 3 ml of centrifuged cerebrospinal fluid (CSF). To evaluate the incremental value of MTB/RIF over a clinically based diagnosis, test accuracy was compared to a clinical score (CS) derived using basic clinical and laboratory information.

Of 204 evaluable patients (of whom 87% were HIV-infected), 59 had definite TBM, 64 probable TBM, and 81 non-TBM. Overall sensitivity and specificity (95% CI) were 62% (48%–75%) and 95% (87%–99%), respectively. The sensitivity of Xpert MTB/RIF was significantly better than that of smear microscopy (62% versus 12%; *p* = 0.001) and significantly better than that of the CS (62% versus 30%; *p* = 0.001; C statistic 85% [79%–92%]). Xpert MTB/RIF sensitivity was higher when centrifuged versus uncentrifuged samples were used (82% [62%–94%] versus 47% [31%–61%]; *p* = 0.004). The combination of CS and Xpert MTB/RIF (Xpert MTB/RIF performed if CS<8) performed as well as Xpert MTB/RIF alone but with a ∼10% reduction in test usage. This overall pattern of results remained unchanged when the definite and probable TBM groups were combined. Xpert MTB/RIF was not useful in identifying TBM among HIV-uninfected individuals, although the sample was small. There was no evidence of PCR inhibition, and the limit of detection was ∼80 colony forming units per millilitre. Study limitations included a predominantly HIV-infected cohort and the limited number of culture-positive CSF samples.

**Conclusions:**

Xpert MTB/RIF may be a good rule-in test for the diagnosis of TBM in HIV-infected individuals from a tuberculosis-endemic setting, particularly when a centrifuged CSF pellet is used. Further studies are required to confirm these findings in different settings.

*Please see later in the article for the Editors' Summary*

## Abstract 2

Vinod Patel and colleagues evaluate the sensitivity and specificity of quantitative PCR using Xpert MTB/RIF for diagnosis of TB meningitis in the high-burden setting of South Africa.

*Please see later in the article for the Editors' Summary*

## Editors' Summary

**Background:**

Worldwide, tuberculosis (TB) is the leading cause of death among people living with HIV. The risk of developing TB is estimated to be 12–20 times greater in people with HIV than in people without HIV. The World Health Organization reported that, in 2011, there were 8.7 million new cases of TB, of which 1.1 million were among people living with HIV. TB infection in people living with HIV is a major problem in sub-Saharan Africa, where up to 80% of individuals infected with TB are also infected with HIV.

TB is caused by a bacterial infection spread through the air from one person to another when the infected person coughs or sneezes, for example. It usually affects the lungs, but it can also affect other parts of the body including the brain, where it leads to meningitis. People with meningitis caused by TB are often seriously ill. Many may develop brain damage, and 30% will die, particularly if they aren't diagnosed quickly and treatment is delayed. TB meningitis is therefore a serious health concern in countries with high rates of HIV and TB co-infection.

**Why Was This Study Done?:**

There is currently no simple test to diagnose TB meningitis. The tests that are available detect only about 50% of cases. They are expensive and practical to use only in a high-tech environment, and are therefore unsuitable for low-income countries.

Recently, a new test has become available to detect TB, known as Xpert MTB/RIF. The test is used to detect the DNA (the molecular biological instructions for each organism) of the bacteria that causes TB. It is accurate at detecting TB lung infection, requires minimal training to operate, and is relatively inexpensive. It is now being used to diagnose TB in countries with high rates of the disease, including South Africa. However, thus far its use has been limited to detecting the TB bacterium in sputum samples (a mixture of saliva and phlegm) from people with a lung infection. The few studies that have assessed whether the test can be used to detect TB meningitis have been small and inconclusive.

This study was carried out to determine whether Xpert MTB/RIF could be used to detect TB bacteria in the cerebrospinal fluid (the fluid that surrounds the brain and spinal cord) in people with TB meningitis. The researchers wanted to find out whether the test would be sensitive (correctly identifying patients with TB meningitis) and specific (correctly identifying patients without TB meningitis). They also wanted to address more practical questions such as how much cerebrospinal fluid needs to be collected and how the sample needs to be processed to ensure accurate results.

**What Did the Researchers Do and Find?:**

The researchers used the Xpert MTB/RIF test to analyze cerebrospinal fluid samples from 204 patients with suspected TB meningitis. These patients were recruited from hospitals in South Africa between January 2008 and December 2011.

Standard diagnostic tests were used to categorize these patients as either definitely having TB meningitis, possibly having TB meningitis, or not having TB meningitis. Among patients infected with HIV, the Xpert MTB/RIF correctly identified 62% of those with TB meningitis and 95% of those without TB meningitis.

The researchers also assessed whether it would be more cost-effective to use the test only for cases where the standard diagnostic procedure was uncertain, i.e., to avoid testing in cases where TB meningitis was very likely following the normal clinical assessment. Based on the researchers' theoretical analysis, this would reduce test use by only about 10%.

**What Do These Findings Mean?:**

This study suggests that Xpert MTB/RIF is a useful diagnostic test for TB meningitis in patients infected with HIV living in areas where there are high levels of TB infection. It is not known how well the test would perform in places where TB levels are low, and the test did not perform well in individuals without HIV, although there were very few of these patients. The Xpert MTB/RIF test correctly identified more positive cases than the other tests used to diagnose TB meningitis, within 24 hours of first seeing a patient. However, the test accuracy was best when the cerebrospinal fluid sample was centrifuged (spinning the sample very fast to concentrate the test material) to achieve the best results. This means additional apparatus would be required, resulting in higher cost and requiring more training. The researchers conclude that this test could still be useful in settings where resources are limited.

There are also important questions that remain unanswered. This study shows only that the Xpert MTB/RIF test is useful in determining that a patient has TB meningitis. It is not useful in determining that a patient does not have TB meningitis. Further research is needed to determine whether the test will be effective in areas with lower rates of TB, as well as whether its use will improve clinical practice and ultimately lead to better outcomes for patients. The hope is that the test will result in more rapid diagnosis and faster treatment, reducing the number of avoidable deaths from TB meningitis.

**Additional Information:**

Please access these websites via the online version of this summary at http://dx.doi.org/10.1371/journal.pmed.1001536.

• This study is further discussed in a *PLOS Medicine* Perspective by David Boulware

• The US National Institutes of Health provides information on TB meningitis

• The World Health Organization provides information on tuberculosis and HIV

• The US Centers for Disease Control and Prevention has a factsheet on HIV and TB

• AIDS.gov also provides information about HIV and TB

## Introduction

There are ∼10 million new cases and 1.7 million deaths from tuberculosis (TB) annually [1]. Although the incidence of TB is decreasing worldwide, it remains a significant cause of morbidity and mortality in sub-Saharan Africa, where, fuelled by the HIV epidemic, it is out of control [1],[2]. In this region, up to 80% of patients infected with TB are co-infected with HIV. Up to 40% of co-infected patients have extrapulmonary TB, and ∼10% of those with extrapulmonary TB have tuberculous meningitis (TBM) [3],[4]. These patients frequently require prolonged admission, they have high morbidity rates due to neuro-pathology, and mortality is substantial (∼30%), particularly if the diagnosis and follow-on therapy are delayed [5]–[8]. Consequently, TBM consumes a disproportionate amount of health care resources. A rapid and affordable confirmatory rule-in and rule-out test still eludes clinical practice.

PCR (polymerase chain reaction) as a diagnostic test for TBM has a sensitivity of ∼50% and a specificity close to 100% [9]. Attempts at improving sensitivity using nested PCR and simultaneous testing using multiple target genes has generally not been fruitful [10]. Given that PCR platforms are generally located in reference laboratories, require technical expertise, are expensive, and are prone to contamination, they are unsuited to resource-limited settings. More recently, however, a closed PCR system, Xpert MTB/RIF (Cepheid), has been developed. Xpert MTB/RIF requires minimal training to operate, is potentially point-of-care, has good accuracy in smear-negative pulmonary TB [11]–[13], and costs ∼US$10 per test, and it has become available as a frontline diagnostic in several high burden countries including South Africa [14]. However, its value has not previously been rigorously evaluated for the diagnosis of TBM. Furthermore, the impact of sample volume and how samples are processed (centrifuged versus uncentrifuged), the limit of detection, the effect of cerebrospinal fluid (CSF)–related PCR inhibition, and the relationship between CSF bacterial load and Xpert MTB/RIF cycle threshold (*C*_T_) values have not been determined. To address these gaps in knowledge, we evaluated the accuracy of Xpert MTB/RIF in an unselected cohort of patients with suspected TBM. To meaningfully ascertain the clinical usefulness and incremental value of the assay relative to pre-test probability (and hence clinical impression), we compared test accuracy to a clinical score (CS) derived from clinical, radiological, and basic laboratory parameters.

## Methods

### Ethics Statement

The study protocol was approved by the biomedical research ethics committees of the University of KwaZulu-Natal and the University of Cape Town.

### Patient Selection

235 consecutive patients with suspected meningitis were prospectively recruited between 1 January 2008 and 31 December 2011. Patients with a meningitic illness who were referred from local district general hospitals were investigated at the tertiary hospital, Inkosi Albert Luthuli Central Hospital, and included in the study.

Written informed consent was obtained from the patient or a close relative. If patients were unable to give consent and a lumbar puncture was clinically indicated, the Head of the Department of Neurology was approached for consent [15]. Patients were clinically assessed, underwent a computerised tomography (CT) scan to exclude contraindications to a lumbar puncture, and had blood drawn for routine tests including a serum fluorescent treponemal antibody test, a venereal disease research laboratory test, an HIV enzyme-linked immunosorbent assay, and a CD4 count. Approximately 15 ml of CSF, obtained by lumbar puncture, was processed for the following tests: microscopy (Gram stain and fluorescent staining for acid-fast bacilli [auramine]); bacterial culture; *Mycobacterium tuberculosis (M.tb.)* culture (Bactec MGIT 960; BD); fungal culture; cryptococcal latex agglutination test; Roche Amplicor Mycobacterium Tuberculosis PCR Test (Roche Diagnostic Systems) (Amplicor PCR); routine chemistry (protein, glucose, chloride); viral PCR for cytomegalovirus, varicella zoster virus, and herpes simplex; venereal disease research laboratory test; fluorescent treponemal antibody test; and test for cysticercus antibodies. An uncentrifuged specimen and, volume permitting, a centrifuged sample of CSF was biobanked for Xpert MTB/RIF analysis. The clinical information recorded included demographic information, duration of symptoms, whether patients were being treated with anti-tuberculous or steroid therapy, HIV status, past history of TB, and history of TB contact.

### Categorisation of Patients

Patients were categorised, based on standardised published diagnostic criteria, as definite TBM if the CSF *M.tb.* culture and/or Amplicor PCR was positive, probable TBM (treated empirically with anti-TB drugs but not meeting the definite TBM criteria), or non-TBM (alternate diagnosis confirmed and response to therapy documented in the absence of anti-TB treatment) [16],[17].

### Amplicor PCR

197 samples were processed by an independent laboratory using the Amplicor PCR kit, for the detection of *M.tb.* This procedure was done as per manufacturer's protocol. Briefly, DNA was extracted from 0.5 ml of CSF using the Roche Magna Pure automated DNA extraction system using the DNA high performance kit. Extracted DNA was then amplified using the biotinylated primers KY18 and KY75 as described in the Amplicor PCR kit protocol. PCR products were detected by the Cobas Amplicor analyser according to the kit protocol.

### Xpert MTB/RIF Assay and Related Bacterial Load Studies

Xpert MTB/RIF is an integrated automated sample-processing and real-time PCR platform developed to simultaneously detect *M.tb.* and rifampicin resistance in a single-use-cartridge hands-free step [18]–[20]. The Xpert MTB/RIF assay consists of two main components, namely, a Xpert MTB/RIF plastic cartridge (containing the liquid sample processing and PCR buffers, and lyophilized real-time PCR reagents with internal sample processing and PCR probe quality controls) and the automated Xpert MTB/RIF machine (which controls the advanced automated portion of the procedure involving the engagement of the fluidics system within the cartridge, automated ultrasound lysis, and the performance of the real-time PCR analysis) [21],[22].

Batched, archived (−70°C) uncentrifuged samples (*n* = 149) and centrifuged and uncentrifuged samples (*n* = 59) were processed at the Lung Infection and Immunity Unit (Department of Medicine, Groote Schuur Hospital, University of Cape Town) for Xpert MTB/RIF analysis. Samples were stored at −70°C, for ethical reasons, for 4 to 6 wk prior to analysis. Both centrifuged and uncentrifuged samples were archived only later in the study when it was decided to test the effect of centrifugation on test accuracy. The laboratory technicians performing the CSF culture, Xpert MTB/RIF assay, and Amplicor PCR were blinded to all participant details.

Samples were prepared for Xpert MTB/RIF according to the manufacturer's instructions [21]. Briefly, the frozen, unprocessed samples were thawed and immediately processed. The CSF/sample reagent mixture was shaken and incubated at room temperature for a total of 15 min, with a second shake occurring at 10 min. 2 ml of the digested mixture was then transferred to the Xpert MTB/RIF cartridge. The automated steps of the procedure were initiated by placing the loaded assay cartridge into the Xpert MTB/RIF instrument module and then selecting the “*M. tuberculosis*-Rif” automated detection test option from the included software. The test was started within 30 min of adding the sample to the cartridge.

Results were interpreted using the automated software. The data analysis algorithms report (1) “*M.tb.* detected” if the *M.tb.* target DNA (*rpoB*) region was detected or (2) “*M.tb.* not detected” if the *M.tb.* target DNA (*rpoB*) region was not detected. If *M.tb.* was detected, the results are further categorised into (1) “RIF-resistance detected” (if a mutation in the *rpoB* gene was detected) or (2) “RIF-resistance not detected” (if no mutation was detected in the *rpoB* region). The detailed principle of the procedure, steps of the automated assay protocol, and full details of the diagnostic algorithms and threshold are described in the manufacturer's package insert [21]. In the initial period (up to 31 January 2011) only 1 ml of uncentrifuged CSF was obtained for Xpert MTB/RIF testing from 149 patients with suspected TBM. From 1 February 2011 onward, to evaluate the impact of centrifugation, a ∼3-ml centrifuged pellet (3,000*g* for 15 min) was obtained from 59 patients with suspected TBM, and resuspended in 1 ml of phosphate-buffered saline. In this latter period, if enough CSF was obtained, both a 1-ml uncentrifuged and 3-ml centrifuged sample were evaluated. Thus, either a 1-ml or 3-ml sample, or both, was processed for Xpert MTB/RIF in 208 patients with suspected TBM. Data were analysed according to HIV status. Included in Text S1 is the detailed method of CSF processing by Xpert MTB/RIF.

We further developed a CS and tested whether Xpert MTB/RIF added diagnostic value above pre-test probabilities when using basic clinical and laboratory values.

Preliminary experiments were performed to determine the detection limit for Xpert MTB/RIF. Patients with motor neuron disease provided written consent for CSF collection. The CSF obtained was spiked with serial dilutions of *M.tb.* (H37Rv). *C*_T_ values were correlated with bacterial load (Bactec MGIT 960 time to culture positivity [TTP]), and PCR inhibition was evaluated by comparing the *C*_T_ value of the internal positive control (IPC; *Bacillus globigii*) in CSF to that in sputum samples obtained from a previously described reference cohort [13]. In those with Xpert MTB/RIF-positive samples, we also compared TTP between those who had an uncentrifuged (1 ml) and those who had a centrifuged (3 ml) Xpert MTB/RIF test performed.

### Development of the Clinical Score

By applying univariable and multivariable logistic regression analysis to clinical and readily available imaging and basic laboratory parameters, we developed a CS and further assessed whether Xpert MTB/RIF had incremental value over the CS. To develop the CS, factors significantly associated with definite TBM (*p*<0.05) in HIV-infected individuals were identified from a multiple logistic regression model, and scores proportionally weighted to the level of significance were assigned. We chose a cut point with a high specificity, as we required a good rule-in test, and so that the performance of the clinical assessment was directly comparable to the performance of the diagnostic assays under study. To determine whether this strategy could reduce test usage in resource-limited settings, an analysis was also conducted to determine the net reduction in test usage if the CS was combined with Xpert MTB/RIF (i.e., Xpert MTB/RIF performed only if the CS was below a rule-in threshold).

### Statistical Analysis

The demographic and clinical characteristics of subgroups (definite TBM, probable TBM, or non-TBM patients, or by test type) were compared using Fisher's exact test for categorical variables and Wilcoxon's rank sum test (two groups; Tables 1 and 2) or the Kruskal-Wallis test (three groups; Table S1) for the continuous variables. Wilcoxon's rank sum test was also used to compare TTP. Factors significantly associated with definite TBM, *p*<0.05, were identified from a multiple logistic regression model. Rounded regression coefficients were used to create an ordinal CS for each patient. A cut-off point at the 90th percentile of the frequency distribution of the scale was selected. Using this cut-off, patients were classified into positive and negative, which forms the dichotomous classification of the CS [23]. Patients negative on the CS were then tested using Xpert MTB/RIF. Differences in specificity and sensitivity between uncentrifuged Xpert MTB/RIF and centrifuged Xpert MTB/RIF tests, differences between Xpert MTB/RIF tests and the CS, and, finally, the incremental value of adding the Xpert MTB/RIF test to the CS were determined using McNemar's Chi square test where tests were repeated on the same sample. Culture and/or Amplicor PCR were used as the gold standard. A two-sample Z test was used where the above two sample groups being compared differed. Data were analysed using Stata version 11 (Statacorp).

**Table 1:** Clinical and cerebrospinal fluid data in HIV-infected patients with definite TBM (liquid culture or Amplicor PCR positive; *n* = 55) and non-TBM disease (culture negative and no anti-TB treatment given; *n* = 70).

Characteristic	Definite TBM, *n* = 55	Non-TBM, *n* = 70	*p*-Value^a^
**Clinical characteristics**			
Mean age in years (standard deviation)	33 (8.9)	34 (8.8)	0.8
Age <36 y/≥36 y^b^	37/18 (67/33)	44/26 (63/37)	0.6
Sex male/female	25/30 (45/55)	17/53 (24/76)	0.01
Ethnic group BA/M/E/I^c^	55/0/0/0 (100/0/0/0)	70/0/0/0 (100/0/0/0)	na
HIV status positive/negative	55/0 (100/0)	70/0 (100/0)	na
Previous TB yes/no/unknown	13/37/5 (23/67/9)	32/36/2 (46/51/3)	0.02
TB contact within last 2 y yes/no/unknown	15/35/5 (27/64/9)	19/48/3 (27/69/4)	0.5
Duration of illness^b^ <6 d/≥6 d/unknown	9/44/2 (16/80/4)	6/62/2 (9/89/3)	0.7
Steroid treatment yes/no	23/32 (42/58)	9/61 (13/87)	0.001
Cryptococcal latex agglutination test positive yes/no	4/49 (8/92)	32/38 (46/54)	0.001
CD4 cells/µl (IQR)	81.0 (43–140)	136 (54–253)	0.02
**CSF parameters, median (IQR)**			
Lymphocytes (cells/µl)	89 (28–230)	36 (12–104)	0.004
Neutrophils (cells/µl)	65 (20–138)	8 (0–40)	<0.001
Protein (g/l)	1.9 (1.2–2.8)	1.0 (0.8–1.9)	<0.001
CSF glucose (mmol/l)	1.2 (1.0–1.8)	2.2 (1.5–2.7)	<0.001
CSF:serum glucose ratio	0.2 (0.2–0.3)	0.4 (0.2–0.5)	<0.001
Lymphocyte:total cell ratio	0.5 (0.3–0.8)	0.9 (0.5–1.00)	0.005

Values are number (percent) unless otherwise indicated.

a Categorical variables were compared using Fisher's exact test, and numeric variables using Wilcoxon's rank sum test.

b This cut point was chosen based on criteria derived by Thwaites et al. [35].

c BA, Black African; M, mixed race; E, European; I, Indian.

na, not applicable.

**Table 2:** Clinical and CSF data in HIV-uninfected patients with definite TBM (liquid culture or Amplicor PCR positive; *n* = 4) and non-TBM disease (culture negative and no anti-TB treatment given; *n* = 11).

Characteristic	Definite TBM, *n* = 4	Non-TBM, *n* = 11	*p*-Value^a^
**Clinical characteristics**			
Mean age in years (standard deviation)	30 (10.8)	28 (16.4)	0.9
Age <36 y/≥36 y^b^	3/1 (75/25)	8/3 (73/28)	0.9
Sex male/female	3/1 (75/25)	9/2 (82/18)	0.8
Ethnic group BA/M/E/I^c^	3/1/0/0 (75/25/0/0)	9/0/0/2 (82/0/0/18)	0.3
HIV status positive/negative	0/4 (0/100)	0/11 (0/100)	na
Previous TB yes/no/unknown	0/4/0 (0/100/0)	0/9/2 (0/82/18)	0.4
TB contact within last 2 y yes/no/unknown	0/4/0 (0/100/0)	1/9/1 (9/82/9)	0.7
Duration of illness^b^ <6 d/≥6 d/unknown	0/4/0 (0/100/0)	5/4/2 (45/36/18)	0.03
Steroid treatment yes/no	1/3 (25/75)	2/9 (18/82)	0.8
Cryptococcal latex agglutination test positive yes/no	0/4 (0/100)	0/11 (0/100)	na
CD4 cells/µl (IQR)	246 (120–426)	678 (344–735)	0.07
**CSF parameters, median (IQR)**			
Lymphocytes (cells/µl)	128 (77–362)	14 (4–38)	0.05
Neutrophils (cells/µl)	73 (40–136)	8 (0–278)	0.3
Protein (g/l)	1.2 (1.2–1.4)	0.7 (0.5–1.4)	0.2
CSF glucose (mmol/l)	0.9 (0.6–1.0)	3.4 (2.5–4.1)	0.005
CSF:serum glucose ratio	0.2 (0.1–0.2)	0.6 (0.5–0.7)	0.005
Lymphocyte:total cell ratio	0.6 (0.4–0.8)	0.6 (0.02–1.0)	0.9

Values are number (percent) unless otherwise indicated.

a Categorical variables were compared using Fisher's exact test, and numeric variables using Wilcoxon's rank sum test.

b This cut point was chosen based on criteria derived by Thwaites et al. [35].

c BA, Black African; M, mixed race; E, European; I, Indian.

na, not applicable.

## Results

### Clinical and Laboratory Features

**Figure 1:**
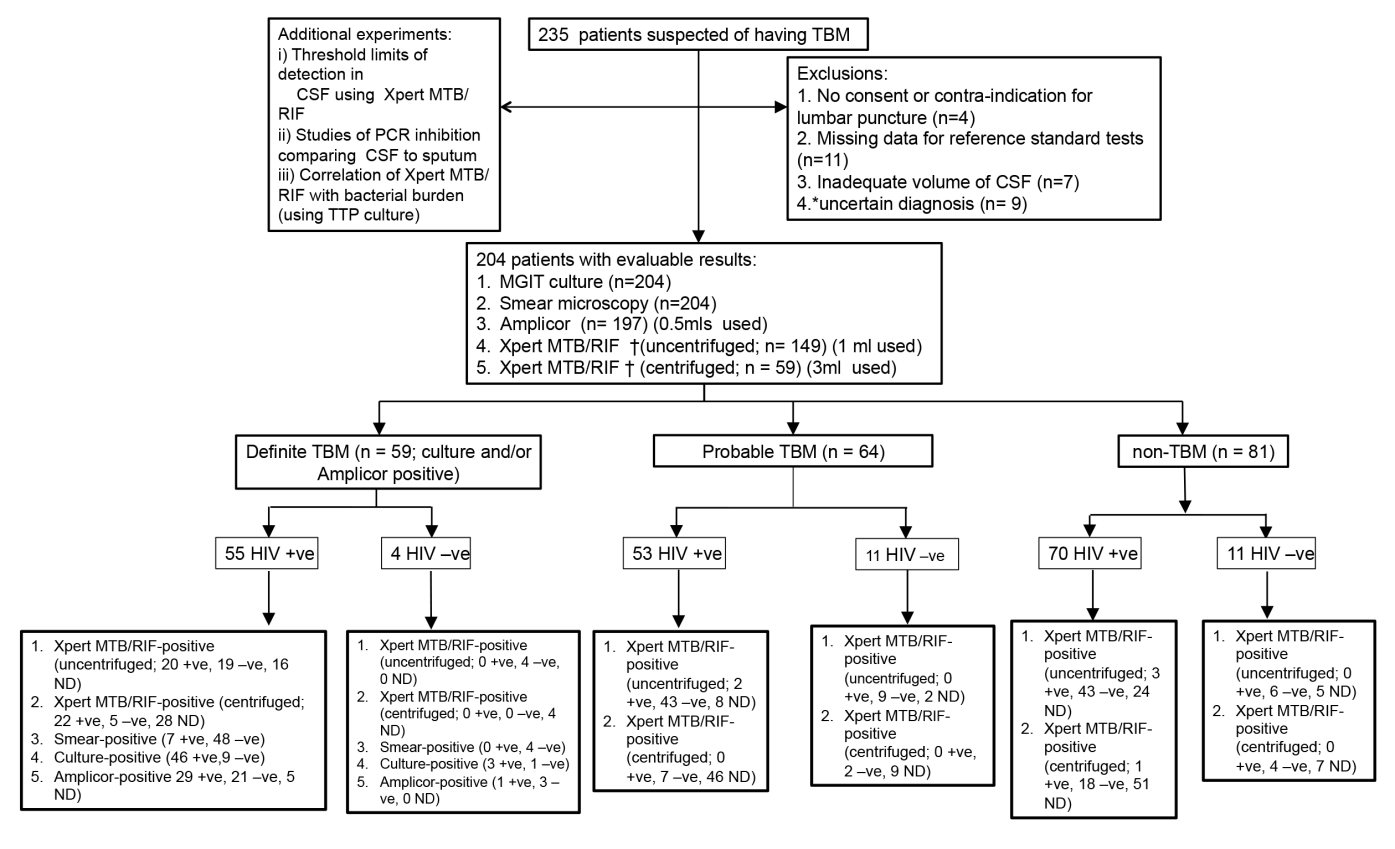
Summary flow chart of patient recruitment and diagnostic testing performed. MGIT, Bactec MGIT 960; ND, not done; +ve, positive; −ve, negative. * These patients could not be clearly categorised as definite TBM, probable TBM, or non-TBM (e.g., reference negative and lost to follow-up, and without initiation of TB treatment). † Note that the uncentrifuged and centrifuged Xpert MTB/RIF groups include 12 patients who had both processes done, i.e., paired samples.

Of the 235 recruited patients, there were 31 exclusions (see Figure 1 for details, including study overview). Of the remaining 204 patients, 59 had definite TBM (seven were smear positive; the rest were culture and/or Amplicor PCR positive), 64 had probable TBM, and 81 had a non-TBM disease. 179 (87%) of participants were HIV-infected, and their median CD4 count was 141 (interquartile range [IQR]: 66–284) The non-TBM group (*n* = 81) comprised the following breakdown of diagnoses (number): cryptococcal meningitis (36), viral meningitis (25), mucormycosis (1), acute bacterial meningitis (7), malignant meningitis (3), cysticercal meningitis (1), neurosyphilis (2), parameningeal focus (1), and other (5). There were 50 HIV-infected patients in the definite-TBM group, 53 HIV-infected patients in the probable-TBM group, and 70 HIV-infected patients in the non-TBM group.

A detailed breakdown of CSF processing results is outlined in Figure 1. Table 1 shows the demographic and laboratory data when comparing the HIV-infected definite-TBM and non-TBM groups. Both groups were comparable, except for the use of steroid therapy and cryptococcal latex agglutination test negativity (Table 1). The CD4 count was significantly higher in the non-TBM group (*p* = 0.02). Furthermore, all routine CSF parameters—including neutrophil and lymphocyte counts, protein level, glucose level, and CSF:blood glucose ratios—were significantly higher in the definite-TBM group. The same pattern of results was seen in HIV-uninfected individuals, except for the lymphocyte:total cell ratio, for which the *p*-value was 0.9 (shown in Table 2). In the definite-TBM group, 73% of patients were being treated with a fixed-dosed combination of first-line anti-TB treatment with a median duration of treatment of 2.5 d (IQR: 0–4) at the time of initial assessment. The non-TBM group had treatment appropriate to the diagnosis considered and were not on TB therapy. A comparison of demographic data between patients who had uncentrifuged, centrifuged, or both Xpert MTB/RIF tests performed showed no differences (Table S1). Text S2 reflects the percentage of TBM patients classified according the Medical Research Council grading for severity. This article fulfils the STARD requirements (Text S3).

### Multivariable Regression Analysis Identifying Clinical and CSF Parameters That Were Predictive of TBM in HIV-Infected Patients

Factors predictive for TBM are shown in Table 3. Applying receiver operating characteristic curve analysis, a clinical prediction rule was developed where a rule-in score of CS≥8 predicted TBM with a sensitivity of 30% (95% CI 14%–50%) and a specificity of 100% (95% CI 85%–100%) (C statistic 85% [95% CI: 79%–92%]). When centrifuged Xpert MTB/RIF was added to the CS, sensitivity improved to 89% (95% CI 71%–98%), *p* = 0.001. Detailed accuracy data are shown in Table 4.

**Table 3:** Multivariable regression analysis identifying factors predictive for TBM and related derivation of the clinical score for HIV-infected patients.

Parameter	Odds Ratio (95% CI)	*p*-Value	B-Coefficient	Score
**Cryptococcal latex agglutination test**				
Negative	17.6 (4.7–66.4)	<0.001	2.9	3
Positive	1		0	0
**CSF:plasma glucose ratio**				
≤0.2	4.6 (1.5–14.2)	0.009	1.52	2
>0.2	1		0	0
**CD4 count**				
≤200 cells/µl	8.6 (2.4–30.8)	0.001	2.2	2
>200 cells/µl	1		0	0
**Lymphocyte count**				
>200 cells/µl	8.0 (1.9–34.0)	0.005	2.08	2
≤200 cells/µl	1		0	0
**Hydrocephalus**				
Yes	5.8 (1.7–19.2)	0.004	1.80	2
No	1		0	0

**Table 4:** Performance outcomes of Xpert MTB/RIF (overall, uncentrifuged, and centrifuged), smear microscopy, clinical score, and a combination of Xpert MTB/RIF and clinical score.

Test Specifics	Sensitivity (95% CI) [*n*]	Specificity (95% CI) [*n*]	PPV (95% CI) [*n*]	NPV (95% CI) [*n*]	Agreement (95% CI) [*n*]
**Definite TBM versus non-TBM** a					
Smear microscopy	13%(5–25)^b^[7/55]	100%(95–100)[70/70]	100%(59–100)[7/7]	59%(50–68)[70/118]	62%(53–70)[77/125]
Xpert MTB/RIF (all samples, whether uncentrifuged or centrifuged)	67%^b^(53–79)[36/54]	94%(85–98)[61/65]	90%(76–97)[36/40]	77%(66–86)[61/79]	82%(73–88)[97/119]
Uncentrifuged Xpert MTB/RIF	51%(35–68)^b^^,^ c[20/39]	94%(82–99)[43/46]	87%(66–97)[20/23]	69%(56–80)[43/62]	74%(64–83)[63/85]
Centrifuged Xpert MTB/RIF	82%(62–94)^b^^,^ c[22/27]	95%(74–100)[18/19]	96%(78–100)[22/23]	78%(56–93)[18/23]	87%(74–95)[40/46]
CS alone (score≥8)	30%(14–50)^b^^,^ d[8/27]	100% (82–100) [19/19]	100%(63–100)[8/8]	50%(33–67)[19/38]	59%(43–73)[27/46]
CS plus centrifuged Xpert MTB/RIF (only done if CS<8)	89%(71–98)^d^[24/27]	95% (74–100) [18/19]	96%(80–100)[24/25]	86%(64–97)[18/25]	91%(79–98)[42/46]
**Combined definite and probable TBM versus non-TBM** e					
Smear microscopy	6%(3–13)[7/108]	100%(95–100)[70/70]	100%(59–100)[7/7]	41%(34–49)[70/171]	43%(36–51)[77/178]
Xpert MTB/RIF (all samples, whether uncentrifuged or centrifuged)	36%(27–46)[38/106]	94%(85–98)[61/65]	91%(77–97)[38/42]	47%(38–56)[61/129]	58%(50–65)[99/171]
Uncentrifuged Xpert MTB/RIF	26%^f^(17–37)[22/84]	94%(82–99)[43/46]	88%(69–98)[22/25]	41%(32–51)[43/105]	50%^g^(41–59)[65/130]
Centrifuged Xpert MTB/RIF	65%^f^(47–80)[22/34]	95%(74–100][18/19]	96%(78–100)[22/23]	60%(41–77)[18/30]	76%^g^(62–86)[40/53]

Sensitivity, specificity, PPV, NPV, and CS are expressed as percentages.

a Performance outcomes when definite TBM is compared with non-TBM (liquid culture or Amplicor PCR positivity for *M.tb.* served as a reference standard).

b Represents a comparison of sensitivity between microscopy and centrifuged Xpert MTB/RIF, *p*≤0.001; microscopy and uncentrifuged Xpert MTB/RIF, *p*≤0.001; microscopy and CS alone, *p* = 0.8; microscopy and CS plus centrifuged Xpert MTB/RIF, *p*≤0.001; microscopy with Xpert MTB/RIF (regardless of centrifugation or volume), *p*≤0.001.

c Represents a comparison of Xpert MTB/RIF sensitivity between centrifuged and uncentrifuged samples, *p* = 0.004.

d Represents a comparison of sensitivity between CS alone and CS combined with Xpert MTB/RIF when CS negative using centrifuged samples, *p*≤0.001.

e Performance outcomes when combined definite and probable TBM is compared to non-TBM (liquid culture or Amplicor PCR positivity for *M.tb.* and satisfaction of probable TBM, as defined by Thwaites et al. [16],[17], served as a reference standard).

f Denotes a comparison of sensitivities between uncentrifuged and centrifuged Xpert MTB/RIF, *p*≤0.001.

g Denotes comparison for agreement between uncentrifuged and centrifuged Xpert MTB/RIF, *p*≤0.006.

### Overall Accuracy of Xpert MTB/RIF Using Centrifuged or Uncentrifuged Samples from HIV-Infected Individuals

When the definite-TBM (culture and/or Amplicor PCR positive) and non-TBM groups were used to calculate accuracy, the overall sensitivity and specificity (95% CI) of Xpert MTB/RIF were 67% (53%–79%) and 94 (85%–98%), respectively (Table 4). In HIV-infected individuals, the sensitivity of Xpert MTB/RIF was significantly better than that of microscopy (*p* = 0.001), and the sensitivity of centrifuged samples was significantly better than that of uncentrifuged samples, *p* = 0.0003. When definite TBM combined with probable TBM was compared with non-TBM, regardless of whether the CSF was uncentrifuged (1 ml) or centrifuged (3 ml), the sensitivity and specificity (95% CI) were 36% (27%–46%) and 94% (85%–98%), respectively (Table 4). One patient, who had definite TBM, had an indeterminate Xpert MTB/RIF result. This patient was excluded from the analysis. The HIV-uninfected group had 0% sensitivity for all tests done (Table 5).

**Table 5:** Performance outcomes of Xpert MTB/RIF (overall, uncentrifuged, and centrifuged), smear microscopy, clinical score, and a combination of Xpert MTB/RIF and clinical score.

Test Specifics	Sensitivity (95% CI) [*n*]	Specificity (95% CI) [*n*]	PPV (95% CI) [*n*]	NPV (95% CI) [*n*]	Agreement (95% CI) [*n*]
**Definite TBM versus non-TBM** a					
Smear microscopy	0%(0–60)[0/4]	100%(72–100)[11/11]	0%[0/0]	73%(45–92)[11/15]	73%(45–92)[11/15]
Xpert MTB/RIF (all samples, whether uncentrifuged or centrifuged)	0%(0–60)[0/4]	100%(69–100)[10/10]	0%[0/0]	71%(42–97)[10/14]	71%(42–92)[10/14]
Uncentrifuged Xpert MTB/RIF	0%(0–60)[0/4]	100%(54–100)[6/6]	0%[0/0]	60%(26–88)[6/10]	60%(26–88)[6/10]
Centrifuged Xpert MTB/RIF	0%[0/0]	100%(40–100)[4/4]	0%[0/0]	100%(40–100)[4/4]	100%(40–100)[4/4]
CS alone (score≥8)	0%[0/0]	100%(40–100)[4/4]	0%[0/0]	100%(40–100)[4/4]	100%(40–100)[4/4]
CS plus centrifuged Xpert MTB/RIF (only done if CS<8)	0%[0/0]	100%(40–100)[4/4]	0%[0/0]	100%(40–100)[4/4]	100%(40–100)[4/4]
**Combined definite and probable TBM versus non-TBM** b					
Smear microscopy	0%(0–22)[0/15]	100%(72–100)[11/11]	0%[0/0]	42%(23–63)[11/26]	42%(23–63)[11/26]
Xpert MTB/RIF (all samples, whether uncentrifuged or centrifuged)	0%(0–23)[0/14]	100%(69–100)[10/10]	0%[0/0]	42%(22–63)[10/24]	42%(22–63)[10/24]
Uncentrifuged Xpert MTB/RIF	0%(0–25)[0/13]	100%(54–100)[6/6]	0%[0/0]	32%(13–57)[6/19]	32%(13–57)[6/19]
Centrifuged Xpert MTB/RIF	0%(0–84)[0/2]	100%(40–100][4/4]	0%[0/0]	67%(22–96)[4/6]	67%(22–96)[4/6]

a Performance outcomes when definite TBM is compared with non-TBM (liquid culture or Amplicor PCR positivity for *M.tb.* served as a reference standard).

b Performance outcomes when combined definite and probable TBM is compared to non-TBM (liquid culture or Amplicor PCR positivity for *M.tb.* and satisfaction of probable TBM, as defined by Thwaites et al. [16],[17], served as a reference standard).

### Xpert MTB/RIF Using Uncentrifuged CSF

There were 149 samples processed for Xpert MTB/RIF using 1 ml of uncentrifuged CSF. Details of the breakdown of results are shown in Figure 1. The sensitivity and specificity (95% CI) of uncentrifuged Xpert MTB/RIF were 51% (35%–68%) and 94% (82%–99%), respectively (see Table 4). There were two patients infected with TBM resistant to rifampicin. Resistance was confirmed by drug sensitivity testing. Sensitivity in the uncentrifuged samples was 26% (17%–37%), and specificity was 94% (82%–99%). Accuracy data for the combined definite- and probable-TBM groups compared to the non-TBM group are shown in Table 4. Data from HIV-uninfected individuals (0% sensitivity for all tests) are shown in Table 5.

### Xpert MTB/RIF Using Centrifuged CSF

There were 59 samples prospectively tested via Xpert MTB/RIF using 3 ml of centrifuged CSF. Details of the breakdown of results are shown in Figure 1. In the HIV-infected group, the sensitivity and specificity (95% CI) of centrifuged Xpert MTB/RIF were 82% (62%–94%) and 95% (74%–100%), respectively (see Table 4). When the combined definite- and probable-TBM groups were compared with the non-TBM group, the sensitivity and specificity (95% CI) of centrifuged Xpert MTB/RIF improved relative to the uncentrifuged samples to 65% (47%–87%) and 96% (78%–100%), respectively. Further accuracy data when the combined definite- and probable-TBM groups were compared to non-TBM is shown in Table 4. The HIV-uninfected group showed a sensitivity of 0% across all tests (Table 5).

### Influence of Prevalence and Impact of Test Usage

As positive predictive value (PPV) and negative predictive value (NPV) are strongly influenced by prevalence of disease, we included an analysis of how the CS, when hypothetically applied to a cohort of 100 patients with suspected TBM, would perform, and how many Xpert MTB/RIF tests would potentially be unnecessary if the CS were applied prior to performing the centrifuged Xpert MTB/RIF test. True hospital prevalence of TBM in South Africa is not known. A hypothetical prevalence of 59% (based on the number of patients with definite plus probable TBM in this cohort) was assumed for this calculation. When applied to 100 patients with suspected TBM (thus potentially 59 TBM patients), 100 cartridges would have been used, and centrifuged Xpert MTB/RIF would have detected 47 (79.6%) patients. As the hospital prevalence of TBM is variable, we have calculated the number of tests saved when assuming a hospital prevalence varying from 10% to 59% (shown in Table 6). In an alternative strategy using the 59% prevalence, CS (with CS≥8 indicating TBM) would have categorised 17 patients as having TBM. If centrifuged Xpert MTB/RIF were applied to the remainder, a further 34 patients would have been detected. Thus, the combined strategy would detect 51 (80%) patients. CS would thus hypothetically have saved 17 tests, assuming that there were no indeterminate results. Given the bias of selecting prevalence based on definite-TBM and non-TBM cases only, and given that the approximate prevalence in a hospital-based cohort is closer to 30% [24],[25], this strategy would have detected nine patients by CS and a further 17 patients in the remainder by centrifuged Xpert MTB/RIF, i.e., a total of 26 (80%) patients (at a 30% hospital-based prevalence of TBM). Centrifuged Xpert MTB/RIF alone would have detected 24 patients (80%). Thus, CS would have saved nine tests.

**Table 6:** The number of cartridges potentially saved when using CS prior to centrifuged Xpert MTB/RIF testing in a hypothetical cohort of 100 patients with suspected TBM.

Assumed Hospital Prevalence of TBM	Cartridges Used without CS^a^ Being Applied in 100 Patients with Suspected TBM	Number of Patients Identified by CS Alone	Number Identified by Xpert MTB/RIF Alone (No CS Applied)	Number of Patients Identified by CS and Xpert MTB/RIF (When CS<8)	Number of Cartridges Saved
10% (*n* = 10 TBM cases)	100	3/10 (30%)	8/10 (80%)	9/10 (90%)	3/100 (3%)
20% (*n* = 20 TBM cases)	100	6/20 (30%)	16/20 (80%)	17/20 (85%)	6/100 (6%)
30% (*n* = 30 TBM cases)	100	9/30 (30%)	25/30 (83%)	26/30 (87%)	9/100 (9%)
40% (*n* = 40 TBM cases)	100	12/40 (30%)	33/40 (82%)	35/40 (88%)	12/100 (12%)
50% (*n* = 50 TBM cases)	100	15/50 (30%)	41/50 (82%)	44/50 (88%)	15/100 (15%)
59% (*n* = 59 TBM cases)	100	18/59 (30%)	48/59 (81%)	52/59 (88%)	18/100 (18%)

a A CS was generated only to estimate the incremental value of Xpert MTB/RIF over clinical assessment using basic clinical and CSF parameters. The CS was not independently validated.

In summary, applying the CS reduced cartridge usage by 9% with an in-hospital disease prevalence of 30%, and by 33% with a prevalence of ∼60% (outlined in Table 6).

### Testing of Paired Centrifuged and Uncentrifuged Samples

Given limited available CSF volumes, there were only 12 culture-positive samples (note: one patient who was classified as having probable TBM had both 1 ml and 3 ml tested but was excluded from this analysis) that were tested concurrently via Xpert MTB/RIF using both 1 ml of uncentrifuged and 3 ml of centrifuged CSF. The sensitivity was not significantly different in centrifuged versus uncentrifuged samples (58% versus 67%; *p* = 0.6; see Table S2).

### Patients Who Tested MTB Positive by Xpert MTB/RIF but Negative by Culture

There were three patients who were classified in the non-TBM group but were Xpert MTB/RIF positive (two had culture-proven cryptococcal meningitis, and another had biopsy-proven leukemic meningitis). Details are shown in Table 7.

**Table 7:** Characteristics of patients classified as non-TBM and probable TBM who were Xpert MTB/RIF positive.

Patient	HIV Status	CD4 Count (cells/µl)	Neutrophils	Lymphocyte Count (cells/µl)	Protein (g/l)	CSF (Serum) Glucose (mmol/l)	Cryptococcal Latex Agglutination Test	Diagnosis	Outcome
**CSF culture and microscopy negative (categorised as non-TBM)** a									
1	+ve	132	0	10	3.52	2.0 (9.0)	+ve	Cryptococcal meningitis	Died
2	+ve	305	138	20	0.89	0.6 (3.5)	−ve	B cell^b^ lymphoma	Died
3	+ve	350	66	92	2.26	0.8 (6.5)	+ve	Cryptococcal meningitis	Lost to follow-up^c^
**CSF culture negative (categorised as probable TBM)** d									
1	+ve	84	0	2	0.79	0.8 (7.6)	−ve	TBM	Died
2	+ve	97	0	30	4.99	1.2 (9.3)	−ve	TBM	Improved
3	+ve	29	248	48	1.01	1.0 (5.1)	−ve	TBM	Improved
4	+ve	45	60	8	4.40	1.0 (7.5)	−ve	TBM	Died
5	+ve	177	0	18	0.93	3.1 (6.6)	−ve	TBM	Improved
6	+ve	14	286	50	2.93	1.0 (6.1)	−ve	TBM	Improved
7	+ve	426	38	292	2.19	0.7 (5.0)	−ve	TBM	Improved

a Characteristics of patients who were CSF culture and microscopy negative but Xpert MTB/RIF positive (all the patients were classified as non-TBM).

b Confirmed on histology of spinal cord lesion.

c This patient improved in the short term (first 10 d) on anti-fungal treatment but was lost to follow-up after transfer to a peripheral health care facility. Thus, it is uncertain whether he had a dual infection (cryptococcal meningitis and TBM).

d Characteristics of patients who were CSF culture negative but Xpert MTB/RIF positive (all the patients were empirically treated for TB at presentation).

+ve, positive; −ve, negative.

There were seven patients who were classified only as probable TBM even though they were Xpert MTB/RIF positive. All of these patients were HIV-infected, with CD4 counts varying from 18 cells/µl to 350 cells/µl. A significant proportion had atypical CSF changes. Details regarding these patients are shown in Table 7.

### HIV-Uninfected Individuals

There were too few HIV-uninfected patients to generate reliable performance outcome data. However, Xpert MTB/RIF was negative in all four HIV-uninfected, TBM *M.tb.*–infected patients.

### Detection Threshold, Bacterial Load, and Inhibition Studies

**Figure 2:**
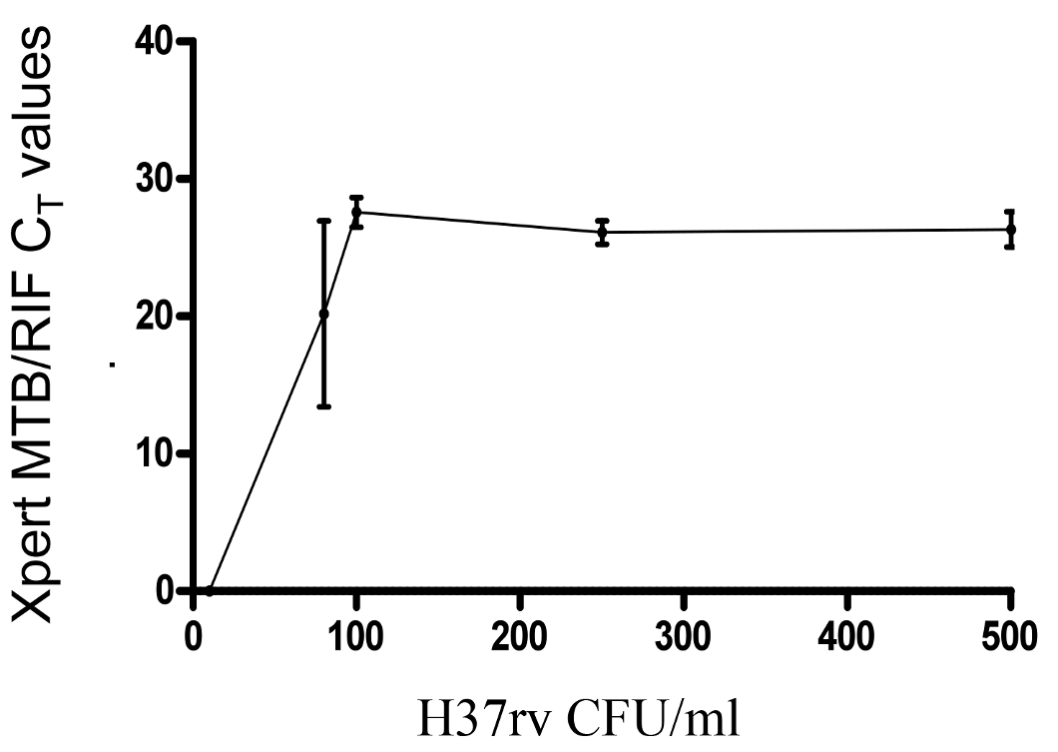
Level of detection of CSF Xpert MTB/RIF for *M. tuberculosis* using serial dilutions (500, 250, 100, 80, and 10 colony forming units per millilitre) of H37Rv. CFU, colony forming units.

**Figure 3:**
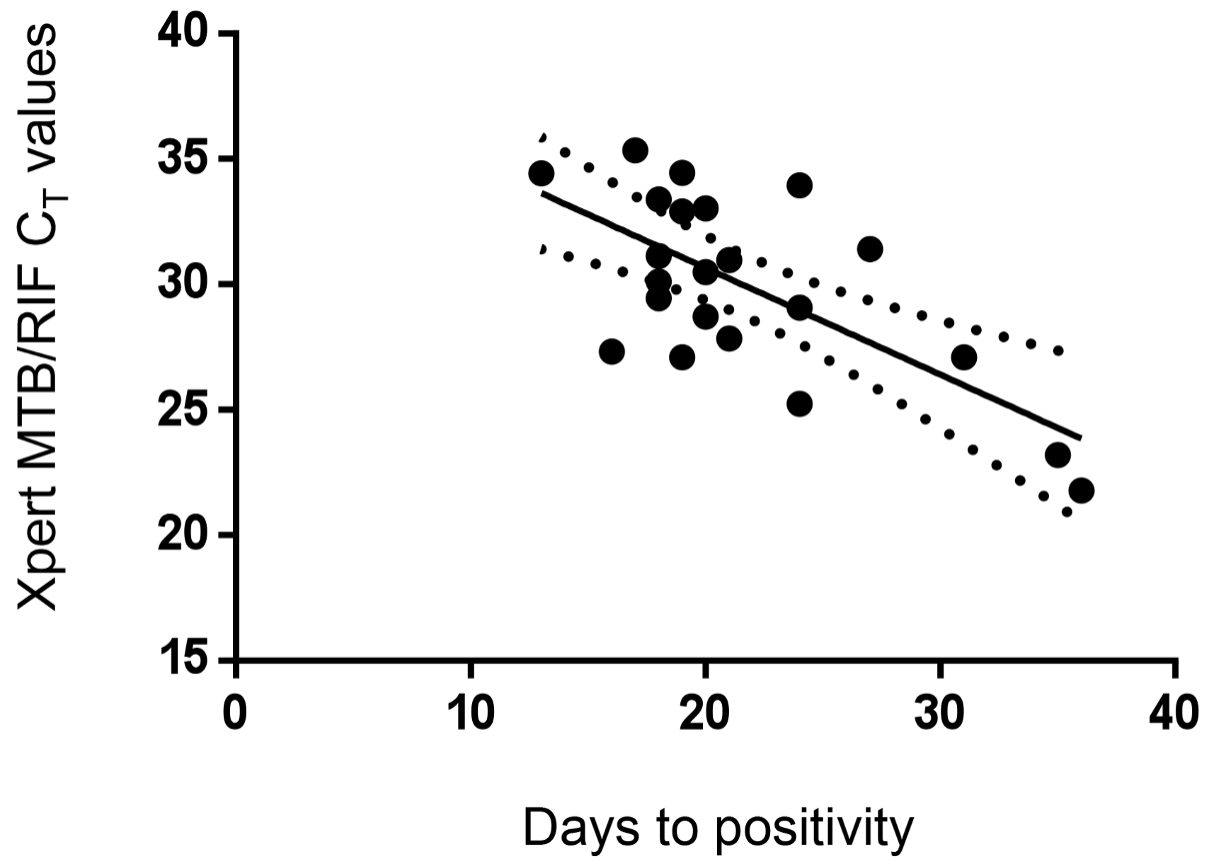
Correlation of CSF Xpert MTB/RIF cycle threshold (*C*_T_) and Bactec MGIT 960 time to positive culture in all samples (both centrifuged and uncentrifuged).

**Figure 4:**
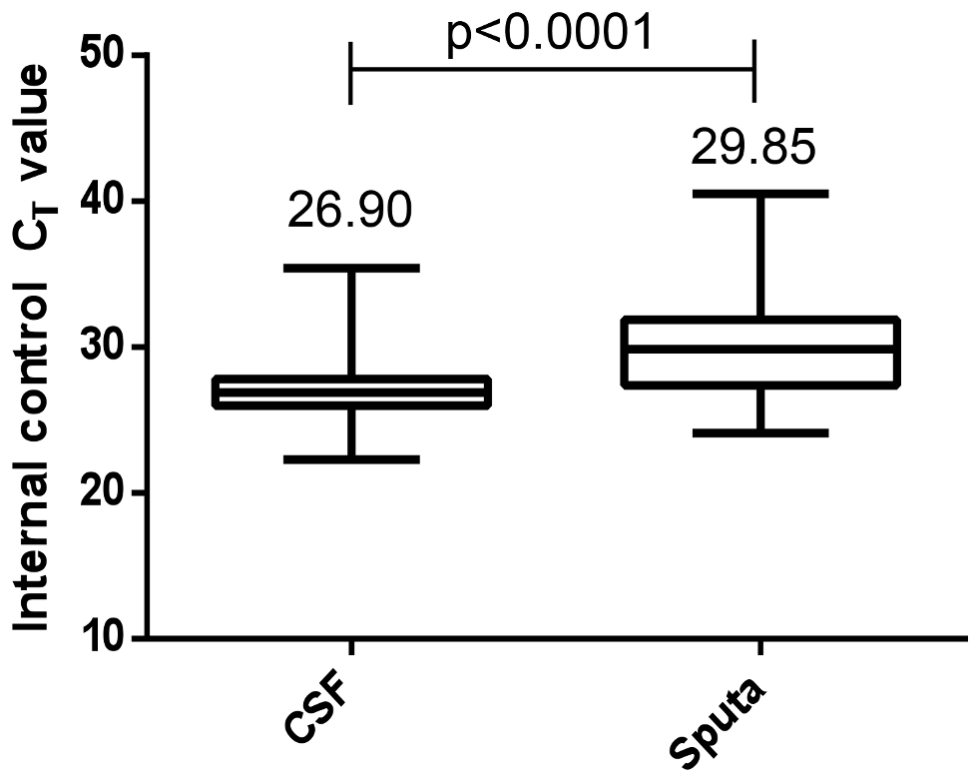
Comparison of PCR inhibition using the comparative internal positive control *C*_T_ values in CSF and sputum (all HIV-infected patients). CSF IPC: median (IQR) *C*_T_ value is 27.2 (range: 27.83–35.4), *n* = 82. Sputa IPC: median (IQR) *C*_T_ value is 29.85 (range: 31.9–40.5), *n* = 238. Comparison between *C*_T_ values for CSF and sputum, *p*≤0.001.

The detection threshold for Xpert MTB/RIF was 80 colony forming units per millilitre (Figure 2). There was no correlation between Xpert MTB/RIF *C*_T_ values and time to positive culture (TTP); (*r* ^2^ = 0.02; *p* = 0.53; see Figure 3). Finally, we examined the degree of relative PCR inhibition by comparing the CSF-specific IPC (*B. globigii*) *C*_T_ values (*n* = 82) to those obtained from sputum samples in a cohort of HIV-infected patients with suspected TB (*n* = 238; *C*_T_ but not IPC data previously published in Blakemore et al. [26]). There was less inhibition using CSF compared to previous published data from Cape Town using sputum (*p*≤0.0001; Figure 4) [13]. Using TTP as a surrogate marker, we compared bacterial load between the centrifuged (*n* = 6) and uncentrifuged (*n* = 9) groups. The median TTP was 19.0 (IQR: 18–24) and 20.0 (IQR: 18–21) d for the uncentrifuged and centrifuged groups, respectively (*p* = 0.9). This may reflect a type 2 statistical error, as the numbers are small.

## Discussion

Although the utility profile and accuracy of Xpert MTB/RIF has been well characterised in sputum samples, there are hardly any data to guide its utility and implementation for TBM. This is critical as the rollout of Xpert MTB/RIF means that quantitative PCR is now available in many high burden settings, and data are urgently required to guide appropriate and relevant usage of this technology in biological fluids other than sputum. That Xpert MTB/RIF performs poorly in fluids from some compartments, e.g., the pleural space, highlights the need for such data [27]. The key findings of this study were as follows: (1) Xpert MTB/RIF is likely a good rule-in test for the diagnosis of TBM in HIV-infected patients; (2) centrifugation of the sample improved sensitivity in this context to almost 80%; (3) among HIV-infected patients, Xpert MTB/RIF performed significantly better than the widely available same-day alternative tests, i.e., smear microscopy, which suggests that prompt diagnosis of TBM is potentially achievable in the majority of patients in this setting; (4) the diagnostic value of Xpert MTB/RIF for HIV-infected patients is clinically meaningful given that it performed significantly better than hypothetical decision-making based on clinical characteristics and basic laboratory data (the CS); and (5) when combined with the CS, Xpert MTB/RIF test usage could be reduced by only a modest ∼10% whilst retaining similar sensitivity and specificity compared to using Xpert MTB/RIF alone. This last finding informs clinical practice in resource-poor settings. Finally, we quantified the limit of detection of the assay, its relationship to bacterial load, and the impact of PCR inhibition. These data require reproduction in HIV-uninfected and non-TB-endemic populations.

There are limited data about Xpert MTB/RIF performance in TBM [28]. Published data include only small numbers of microbiologically proven TBM cases (range of 0 to 23) [29]–[32], often in a case-control design with a non-uniform reference standard, and often CSF-associated data were published as part of a laboratory-based evaluation of extrapulmonary TB samples, usually including samples from countries with low TB prevalence. Furthermore, there are no studies from high burden settings, and technical performance evaluations, including bacterial load studies, threshold level of detection, and impact of PCR inhibition, have hitherto not been undertaken.

Xpert MTB/RIF sensitivity was as high as 80% when a centrifuged CSF sample from an HIV-infected patient was used. This suggests that Xpert MTB/RIF, at least in an HIV-endemic environment, represents a possible new standard of care for the diagnosis of TBM. Sensitivity was considerably better than in previous studies using commercially available or non-standardised PCR tools [9],[32]–[34]. The ostensibly better performance is likely related to a combination of centrifugation (and hence concentration of bacilli) and technical aspects, including a more efficient standardised extraction protocol, fractionation of mycobacteria by a pre-sonication step, and a nested PCR protocol, thus maximising amplification. However, possibly higher bacterial loads in HIV-infected patients may have also played a role. Our findings have practical relevance because they imply that at least 3 ml of CSF should be set aside and centrifuged, and re-suspended in phosphate-buffered saline, before being run on the Xpert MTB/RIF. This high-sensitivity and potentially rapid diagnosis in most cases is likely to benefit HIV-infected patients suspected of having *M.tb.*, as diagnostic and treatment delay is associated with higher mortality [35]–[37]. Impact-related studies are now required to verify this hypothesis. It is noteworthy that a second sample improved sensitivity minimally. These data suggest that, at least in an HIV-endemic setting, using a second cartridge is unlikely to give further benefit. However, larger studies are required to confirm this possibility.

Similar to the findings when using sputum, the level of detection of Xpert MTB/RIF was between 80 and 100 colony forming units per millilitre. This explains the sub-optimal sensitivity of Xpert MTB/RIF compared to culture, where the detection threshold is as low as 1–10 organisms per millilitre [38]. We did not find a correlation between TTP and Xpert MTB/RIF *C*_T_ values, as has been shown in sputum [39]. In contrast to previous PCR-based studies [40],[41], we found that CSF had a minimal inhibitory effect on the PCR reaction when compared to sputum. This may be due to the wash step incorporated into the assay that removes extracellular debris. We did not find a difference in TTP between the Xpert MTB/RIF–positive samples from centrifuged versus uncentrifuged CSF. This may be due to a type two statistical error, as the sample numbers were small.

There were three patients who were culture negative but Xpert MTB/RIF positive, i.e., Xpert MTB/RIF positive in the non-TB group. Our previous work has shown that such cases (Xpert MTB/RIF positive but culture negative) are likely to be true TB positives, and this is corroborated by high specificity obtained in large sputum-based studies where a significant minority of the patients had had previous TB [11]. If these culture-negative Xpert MTB/RIF–positive individuals are hypothetically designated definite-TB cases, then the overall case detection rate improves by a further ∼10%.

The proper and meaningful value of a test lies in its ability to influence patient management through its incremental value over pre-test probability, or to have an impact on decision-making based on logical clinical judgement (based upon clinical features and basic laboratory parameters). We therefore derived a CS, hitherto unavailable for HIV-endemic settings, to evaluate Xpert MTB/RIF utility in clinical practice. Xpert MTB/RIF had significantly better performance outcomes than the clinical prediction rule (using a rule-in cut point, so appropriate comparisons could be made). Furthermore, hypothetically combining the CS with Xpert MTB/RIF resulted only in a modest ∼10% reduction in test usage, but still maintained high sensitivity and specificity. These data suggest that clinical algorithms or scoring systems to limit test usage are unlikely to be significantly useful in resource-poor settings.

There are several limitations of our study. We could not determine the impact of Xpert MTB/RIF (time and proportion of patients initiated on treatment) compared to a smear microscopy/empiric treatment-based strategy given our study design and the fact that management decisions were not based on Xpert MTB/RIF results. However, this was because Xpert MTB/RIF had not yet been endorsed by the World Health Organization when the study commenced, had not been validated for use in CSF, and had been used as a research tool only (thus, for ethical reasons, study samples were evaluated only several weeks later). Although the confidence intervals of some of our estimates are wide (because of limited sample numbers), this is to our knowledge the largest diagnostic study undertaken in TBM (based on the number of microbiologically proven TBM cases; *n* = 59). This reflects the challenge and difficulty in performing such studies in resource-poor settings. It is possible that the Xpert MTB/RIF performs much better in HIV-infected individuals because of a possibly higher bacterial load, and thus our findings need to be confirmed in other settings. Given the small number of HIV-uninfected patients, we were unable to meaningfully compare this sub-group. The CS was developed to assess only incremental value above basic clinical and CSF parameters. The CS and the combination of CS plus Xpert MTB/RIF need prospective and independent validation. The non-significant difference in sensitivity between the paired centrifuged and non-centrifuged samples may reflect a type two statistical error, as the number of culture-positive paired samples was limited. Lastly, there were nine patients who could not be categorised within our defined groups and were excluded from the analysis.

In conclusion, Xpert MTB/RIF may be a good rule-in test for the diagnosis of TBM in HIV-infected individuals in a TB-endemic setting, particularly when a centrifuged CSF pellet is used. A second Xpert MTB/RIF test had minimal incremental benefit. Smear microscopy and the CS, when combined with Xpert MTB/RIF, only modestly minimised test usage in a resource-poor setting. Further studies are now required in non-HIV-endemic settings, and using validated scoring systems, to evaluate the impact of Xpert MTB/RIF on diagnostic accuracy, and morbidity and mortality in patients with TBM.

## Supporting Information

Table S1 **Comparison of demographic data amongst the uncentrifuged, centrifuged, and both centrifuged and uncentrifuged Xpert MTB/RIF test groups.**
(DOCX)

Table S2 **Diagnostic accuracy when comparing centrifuged and uncentrifuged Xpert MTB/RIF in the same patients (all definite TBM,** ***n*** **= 12).**
(DOCX)

Text S1 **Method for processing CSF using Xpert MTB/RIF.**
(DOCX)

Text S2 **Grading of TBM severity.**
(DOCX)

Text S3 **STARD document.**
(DOC)

## Abbreviations

Amplicor PCR: Amplicor Mycobacterium Tuberculosis PCR Test
*C*_T_: cycle threshold
CS: clinical score
CSF: cerebrospinal fluid
CT: computerised tomography
IPC: internal positive control
IQR: interquartile range
*M.tb.*: *Mycobacterium tuberculosis*
NPV: negative predictive value
PPV: positive predictive value
TB: tuberculosis
TBM: tuberculous meningitis
TTP: time to culture positivity
