# Supplementary material for: Diagnostic Accuracy of Quantitative PCR (Xpert MTB/RIF) for Tuberculous Meningitis in a High Burden Setting: A Prospective Study
Source: PLoS Med. 2013 Oct 22;10(10):e1001536. doi: 10.1371/journal.pmed.1001536 (PMC3805498; doi:10.1371/journal.pmed.1001536)
Supplement: Table S1 — Comparison of demographic data amongst the uncentrifuged, centrifuged, and both centrifuged and uncentrifuged Xpert MTB/RIF test groups. (DOCX) [file pmed.1001536.s001.docx]

Table S1. Comparison of demographic data amongst the uncentrifuged, centrifuged and both centrifuged and uncentrifuged GeneXpert^®^ MTB/RIF test groups.

| Characteristic | Uncentrifuged  n (%) | Centrifuged  n (%) | Both  n (%) | P value**^#^** |
| --- | --- | --- | --- | --- |
| **Clinical characteristics** | | | | |
| Mean age (±S.D) | 136**^†^** | 46**^†^** | 13**^‡^** |  |
| Age  < 36 / ≥36 years**^*^** | 92/44 (68/32) | 33/13 (72/28) | 7/6 (54/46) | 0.6 |
| Sex  Male/Female | 49/87 (36/64) | 21/25 (46/54) | 5/8 (38/62) | 0.5 |
| Ethnic group  BA/ M/ E/ I**^†^** | 133/2/0/1 (98/1.5/0/0.5) | 44/0/0/2 (96/0/0/4) | 13/0/0/0 (100/0/0/0) | 0.5 |
| HIV status  P/ N | 118/18 (87/18) | 41/5 (89/11) | 12/1 (92/8) | 0.4 |
| Previous TB  Yes/ No/ Unknown | 34/98/4 (25/72/3) | 15/28/3 (33/61/7) | 6/6/1 (46/46/8) | 0.1 |
| TB contact (within 2 years)  Yes/ No/ Unknown | 31/99/6 (23/73/4) | 10/33/3 (22/72/6) | 7/5/1 (54/38/8) | 0.09 |
| Duration of illness (days)**^*^**  <6 / ≥6 days/Unknown | 20/112/4 (15/82/3) | 4/38/4 (9/83/9) | 2/10/1 (15/77/8) | 0.7 |
| Steroid treatment  Yes/ No | 27/109 (20/80) | 14/32 (30/70) | 6/7 (46/54) | 0.06 |
| Cryptococcal latex agglutination test positive  Yes/ No | 28/108 (21/79) | 4/41 (11/89) | 0/13 (0/100) | 0.1 |
| CD4 cells/μl [IQR] | 173 [72;313] | 90 [45;274] | 104 [72;153] | 0.2 |
| **CSF characteristics median (IQR)** | | | | |
| Lymphocytes (cells/μl) | 39 (10;138) | 65 (20; 160) | 146 (48; 230) | 0.06 |
| Neutrophils (cells/μl) | 22 (6;110) | 26 (2; 70) | 74 (14; 220) | 0.14 |
| Protein g/l | 1.44 (0.93;2.2) | 1.26 (0.6; 2.28) | 2.0 (1.29; 3.6) | 0.05 |
| CSF Glucose mmol/l | 1.8 (1.3; 2.5) | 2.3 (1.1; 2.9) | 1.5 (0.8 1.5) | 0.03 |
| CSF : serum Glucose ratio | 0.30 (0.22;0.42) | 0.29 (0.19; 0.48) | 0.27 (0.13; 0.33) | 0.15 |
| Lymphocytes : total ratio | 0.70 (0.20; 0.95) | 0.80; (0.51; 0.96) | 0.82 (0.37; 0.83) | 0.3 |

* This cut-point was chosen based on criteria derived by Thwaites.*et.al* [[1](#_ENREF_1)]

† BA=Black African, M=mixed race, E=European, I =Indian.

† Note the numbers differ from the main article as the thirteen patients who had both uncentrifuged and centrifuged GeneXpert^®^ MTB/RIF tests done were exclude from both the uncentrifuged and centrifuged groups for separate comparison.

‡ Note that although thirteen patients had both uncentrifuged and centrifuged GeneXpert^®^ MTB/RIF tests done only 12 were include in analysis for accuracy data as they were definite TBM. Here we included the additional patient who was classified as probable TBM.

# Categorical variables were compared using Fisher’s exact test and numeric variables using Wilcoxon Rank sum test.

References

1. Thwaites GE, Chau TT, Stepniewska K, Phu NH, Chuong LV, et al. (2002) Diagnosis of adult tuberculous meningitis by use of clinical and laboratory features. Lancet 360: 1287-1292.
