# Supplementary material for: Diagnostic Accuracy of Quantitative PCR (Xpert MTB/RIF) for Tuberculous Meningitis in a High Burden Setting: A Prospective Study
Source: PLoS Med. 2013 Oct 22;10(10):e1001536. doi: 10.1371/journal.pmed.1001536 (PMC3805498; doi:10.1371/journal.pmed.1001536)
Supplement: Table S2 — Diagnostic accuracy when comparing centrifuged and uncentrifuged Xpert MTB/RIF in the same patients (all definite TBM, n = 12). (DOCX) [file pmed.1001536.s002.docx]

Table S2: Diagnostic accuracy when comparing centrifuged and uncentrifuged GeneXpert^®^ MTB/RIF in the same patients (all definite TBM, n =12**^†^**)

| Category | Sensitivity | Specificity | PPV | NPV | Agreement |
| --- | --- | --- | --- | --- | --- |
| Xpert^®^MTB/RIF Centrifuged | 67%**^*^** (35;90]  [8/12] | 0/0 | 100% (63;100) 8/8 | 0% (0;60) [0/4] | 67% (35;90) [8/12] |
| Xpert^®^MTB/RIF Uncentrifuged | 58%**^*^** (28;85]  [7/12] | 0/0 | 100% (59;100) 7/7 | 0% (0;52) [0/5] | 58% (28;85] [7/12] |

* denotes a comparison of sensitivities between uncentrifuged and centrifuged, Xpert^®^ MTB/RIF p = <0.6.

† Note that one patient not classified as definite was excluded from this comparison.
