# Supplementary material for: Diagnostic Accuracy of Quantitative PCR (Xpert MTB/RIF) for Tuberculous Meningitis in a High Burden Setting: A Prospective Study
Source: PLoS Med. 2013 Oct 22;10(10):e1001536. doi: 10.1371/journal.pmed.1001536 (PMC3805498; doi:10.1371/journal.pmed.1001536)
Supplement: Text S1 — Method for processing CSF using Xpert MTB/RIF. (DOCX) [file pmed.1001536.s003.docx]

Text S1: **Method for processing CSF using Gene Xpert**

**Protocol for Xpert MTB/RIF performed on a resuspended pellet of cerebral spinal fluid**

1. Centrifuge approximately 3 ml of CSF at 3000 *g* for 15 minutes at room temperature (a greater volume may be used, if available)
2. Decant and discard the supernatant
3. Add sterile phosphate buffered saline to a volume of 1 ml
4. Vortex the pellet – phosphate buffered saline mixture so that the pellet is completely suspended.
5. Add 2 ml of Xpert MTB/RIF sample buffer (supplied as part of the test by the manufacturer) and shake for 15 seconds
6. Incubate at room temperature for 15 min, shaking again half way through for 15 s
7. Draw 2 ml of the CSF-PBS-sample buffer suspension into a Pasteur pipette (usually manufacturer supplied) and dispense into the Xpert MTB/RIF cartridge
8. Close the Xpert MTB/RIF cartridge and load it into the machine
9. Continue with the normal Xpert MTB/RIF operating procedure as recommended by the manufacturer

Number and experience of persons doing and reading index tests

One person performed and read the Xpert test (PhD graduate), two persons the culture result (Professor of Microbiology and laboratory technician), and another the Amplicor test (PhD Graduate).
