# Supplementary material for: Diagnostic Accuracy of Quantitative PCR (Xpert MTB/RIF) for Tuberculous Meningitis in a High Burden Setting: A Prospective Study
Source: PLoS Med. 2013 Oct 22;10(10):e1001536. doi: 10.1371/journal.pmed.1001536 (PMC3805498; doi:10.1371/journal.pmed.1001536)
Supplement: Text S2 — Grading of TBM severity. (DOCX) [file pmed.1001536.s004.docx]

Text S2: TBM severity grade and numbers in each group

Severity varied between grades 1 to 3 (1= no focal signs normal level of consciousness, 2= focal signs, 3 = depressed level of consciousness). There were 67% with grade 1 TBM, 26% with grade 2 and 11% with grade 3 TBM. Alternate diagnoses included cryptococcal meningitis, viral meningitis, neoplastic, syphilic, cysticercal and acute bacterial meningitides.
