# Supplementary material for: Diagnostic Accuracy of Quantitative PCR (Xpert MTB/RIF) for Tuberculous Meningitis in a High Burden Setting: A Prospective Study
Source: PLoS Med. 2013 Oct 22;10(10):e1001536. doi: 10.1371/journal.pmed.1001536 (PMC3805498; doi:10.1371/journal.pmed.1001536)

| **Section and Topic** | **Item** |  | **paragraph** |
| --- | --- | --- | --- |
| TITLE/ABSTRACT/ KEYWORDS | 1 | [Identify the article as a study of diagnostic accuracy (recommend MeSH heading 'sensitivity and specificity').](http://www.stard-statement.org/item1_maintext.htm)  This study sought to evaluate the sensitivity and specificity of Xpert Xpert MTB/RIF for the diagnosis of TBM and has the relevant keywords included. | Introduction: paragraph 3,  Abstract: paragraph 1 |
| INTRODUCTION | 2 | [State the research questions or study aims, such as estimating accuracy between tests or across participant groups. These are explicitly covered in the introduction.](http://www.stard-statement.org/item2_maintext.htm) | Introduction: paragraph 3 |
| METHODS |  |  |  |
| *Participants* | 3 | [Describe the study population: The inclusion and exclusion criteria, setting and locations where the data were collected.](http://www.stard-statement.org/item3_maintext.htm)  These are covered in the methods section | Methods: paragraphs 1 and 2 |
|  | 4 | [Describe participant recruitment: Was recruitment based on presenting symptoms, results from previous tests, or the fact that the participants had received the (evaluated) index tests or the (golden) reference standard?](http://www.stard-statement.org/item4_maintext.htm)  235 consecutive patients with suspected meningitis were prospectively recruited between January 2008 and December 2011 | Methods: paragraph 1 |
|  | 5 | [Describe participant sampling: Was the study population a consecutive series of participants defined by the selection criteria in items 3 and 4? If not, specify how participants were further selected.](http://www.stard-statement.org/item5_maintext.htm)  Consecutive series of patients | Methods: paragraph: 1 |
|  | 6 | [Describe data collection: Was data collection planned before the index test and reference standard were performed (prospective study) or after (retrospective study)?](http://www.stard-statement.org/item6_maintext.htm)  Data collection was planned prior to the commencement of the study. The reference standard was predefined. | Methods (categorisation of patient): paragraph 3 |
| *Test methods* | 7 | [Describe the reference standard and its rationale.](http://www.stard-statement.org/item7_maintext.htm)  The reference standard was culture or PCR positivity by Amplicor. This is required for a diagnosis of definite TBM as per consensus guidelines and because maximal sensitivity is required. | Categorisation of patients (Methods) paragraph 3 |
|  | 8 | [Describe technical specifications of material and methods involved including how and when measurements were taken, and/or cite references for index tests and reference standard.](http://www.stard-statement.org/item8_maintext.htm)  This is described in the methods section. See also standard reference below. | Methods (Amplicor, Xpert MTB/RIF): paragraphs 4,5,6 |
|  | 9 | [Describe definition of and rationale for the units, cut-offs and/or categories of the results of the index tests and the reference standard.](http://www.stard-statement.org/item9_maintext.htm)  Cut points were determined using receiver operating characteristic curve analysis to define optimal specificity and PPV as we required a good rule-in test. | Methods (Statistical methods): paragraph 8 |
|  | 10 | [Describe the number, training and expertise of the persons executing and reading the index tests and the reference standard.](http://www.stard-statement.org/item10_maintext.htm)  One person performed and read the Xpert test (PhD graduate), two persons the culture result (Professor of Microbiology and laboratory technician), and another the Amplicor test (PhD Graduate). | Text 1 online supplement |
|  | 11 | [Describe whether or not the readers of the index tests and reference standard were blind (masked) to the results of the other test and describe any other clinical information available to the readers.](http://www.stard-statement.org/item11_maintext.htm)  All readers of the tests were blinded to the clinical information. | Methods (geneXpert assay and related bacterial load studies): Paragraph 2 |
| *Statistical methods* | 12 | [Describe methods for calculating or comparing measures of diagnostic accuracy, and the statistical methods used to quantify uncertainty (e.g. 95% confidence intervals).](http://www.stard-statement.org/item12_maintext.htm)  Outlined on page 12 | Methods (Statistical methods): Paragraph 1 |
|  | 13 | [Describe methods for calculating test reproducibility, if done.](http://www.stard-statement.org/item13_maintext.htm)  Not applicable | Not applicable |
| RESULTS |  |  |  |
| *Participants* | 14 | [Report when study was done, including beginning and ending dates of recruitment.](http://www.stard-statement.org/item14_maintext.htm)  235 consecutive patients with suspected meningitis were prospectively recruited between January 2008 and December 2011 | Results: paragraph 1 |
|  | 15 | [Report clinical and demographic characteristics of the study population (e.g. age, sex, spectrum of presenting symptoms, co morbidity, current treatments, recruitment centers).](http://www.stard-statement.org/item15_maintext.htm)  All patients were referred and recruited at Inkosi Albert Luthuli central hospital. Their mean ages were 33 years, almost all were HIV positive with presentations suggesting meningitis (headache, neck stiffness, confusion, arachnoiditis). | Results: paragraph 1 |
|  | 16 | [Report the number of participants satisfying the criteria for inclusion that did or did not undergo the index tests and/or the reference standard; describe why participants failed to receive either test (a flow diagram is strongly recommended).](http://www.stard-statement.org/item16_maintext.htm) | Figure 1 |
| *Test results* | 17 | [Report time interval from the index tests to the reference standard, and any treatment administered between.](http://www.stard-statement.org/item17_maintext.htm)  The time interval varied between several weeks to several months as tests were batched and processed for Gene Xpert. | Methods (GeneXpert assay and related bacterial load studies) paragraph 2 |
|  | 18 | [Report distribution of severity of disease (define criteria) in those with the target condition; other diagnoses in participants without the target condition.](http://www.stard-statement.org/item18_maintext.htm)  Severity varied between grades 1 to 3 (1=no focal signs normal level of consciousness, 2=focal signs, 3=depressed level of consciousness). There were 67% with grade 1 TBM, 26% with gre 2 and 11% with grade 3 TBM. Alternate diagnoses included cryptococcal meningitis, viral meningitis, neoplastic, syphilic, cysticercal and acute bacterial meningitides. | Online supplement: text 2 |
|  | 19 | [Report a cross tabulation of the results of the index tests (including indeterminate and missing results) by the results of the reference standard; for continuous results, the distribution of the test results by the results of the reference standard.](http://www.stard-statement.org/item19_maintext.htm)  This is done in the paper.  There was one indeterminate result who was culture positive and was excluded from the analysis. | Results  (Overall accuracy of Xpert MTB/RIF): paragraph 1 |
|  | 20 | [Report any adverse events from performing the index tests or the reference standard.](http://www.stard-statement.org/item20_maintext.htm)  There were no adverse events from performing the index test. | NA |
| *Estimates* | 21 | [Report estimates of diagnostic accuracy and measures of statistical uncertainty (e.g. 95% confidence intervals).](http://www.stard-statement.org/item21_maintext.htm)  This is included in the paper | Results (overall, uncentrifuged and centrifuged Xpert MTB/RIF): All paragraph 1 in each section |
|  | 22 | [Report how indeterminate results, missing responses and outliers of the index tests were handled.](http://www.stard-statement.org/item22_maintext.htm)  There were one indeterminate results which were not included in the analysis. The outcome of the tests was binary (pos/neg) so there were no outliers | Results  (Overall accuracy of Xpert MTB/RIF): paragraph |
|  | 23 | [Report estimates of variability of diagnostic accuracy between subgroups of participants, readers or centers, if done.](http://www.stard-statement.org/item23_maintext.htm)  All tests were done on a single machine at a single site. The procedures were automated with an objective result that did not require any subjective interpretation. No estimates of variability were required | NA |
|  | 24 | [Report estimates of test reproducibility, if done.](http://www.stard-statement.org/item24_maintext.htm)  Not applicable | NA |
| DISCUSSION | 25 | [Discuss the clinical applicability of the study findings.](http://www.stard-statement.org/item25_maintext.htm)  The study applies to patients suspected of tuberculous meningitis who are HIV positive from a TB endemic population. | Discussion: Paragraphs 1-6 |

1. Thwaites G, Fisher M, Hemingway C, Scott G, Solomon T, et al. (2009) British Infection Society guidelines for the diagnosis and treatment of tuberculosis of the central nervous system in adults and children. J Infect 59: 167-187.


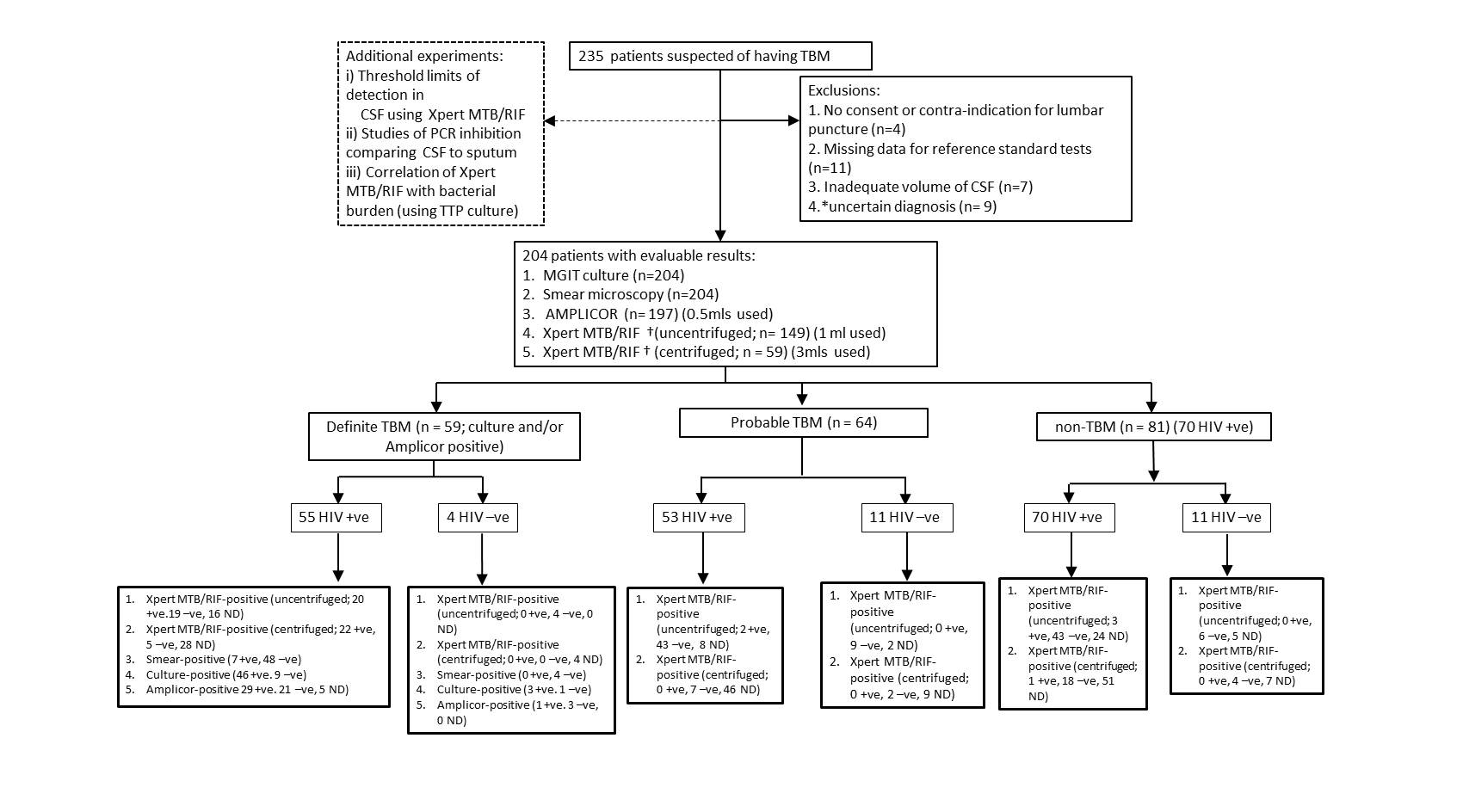

Supplement: Text S3 — STARD document. (DOC) [file pmed.1001536.s005.doc]
